# 102 Longitudinal Changes in Lingual and Intestinal Microbiota in a Swine Burn Model

**DOI:** 10.1093/jbcr/irad045.075

**Published:** 2023-05-15

**Authors:** Timothy Horseman, Andrew Frank, Bonnie Carney, Lauren Moffatt, Jeffrey Shupp, Saira Nisar, John Keyloun, David Burmeister

**Affiliations:** Uniformed Services University of the Health Sciences, Bethesda, Maryland; Uniformed Services University of the Health Sciences, Bethesda, Maryland; Firefighters' Burn and Surgical Research Laboratory, Medstar Health Research Institute, Georgetown University Medical Center, Washington, DC; Medstar Health, Washington DC, DC; Firefighters' Burn and Surgical Research Laboratory, MedStar Health Research Institute, Georgetown University School of Medicine, Washington, DC; Medstar Health, Washington DC, DC; Medstar Health, Washington DC, DC; Uniformed Services University of the Health Sciences, Bethesda, Maryland; Uniformed Services University of the Health Sciences, Bethesda, Maryland; Uniformed Services University of the Health Sciences, Bethesda, Maryland; Firefighters' Burn and Surgical Research Laboratory, Medstar Health Research Institute, Georgetown University Medical Center, Washington, DC; Medstar Health, Washington DC, DC; Firefighters' Burn and Surgical Research Laboratory, MedStar Health Research Institute, Georgetown University School of Medicine, Washington, DC; Medstar Health, Washington DC, DC; Medstar Health, Washington DC, DC; Uniformed Services University of the Health Sciences, Bethesda, Maryland; Uniformed Services University of the Health Sciences, Bethesda, Maryland; Uniformed Services University of the Health Sciences, Bethesda, Maryland; Firefighters' Burn and Surgical Research Laboratory, Medstar Health Research Institute, Georgetown University Medical Center, Washington, DC; Medstar Health, Washington DC, DC; Firefighters' Burn and Surgical Research Laboratory, MedStar Health Research Institute, Georgetown University School of Medicine, Washington, DC; Medstar Health, Washington DC, DC; Medstar Health, Washington DC, DC; Uniformed Services University of the Health Sciences, Bethesda, Maryland; Uniformed Services University of the Health Sciences, Bethesda, Maryland; Uniformed Services University of the Health Sciences, Bethesda, Maryland; Firefighters' Burn and Surgical Research Laboratory, Medstar Health Research Institute, Georgetown University Medical Center, Washington, DC; Medstar Health, Washington DC, DC; Firefighters' Burn and Surgical Research Laboratory, MedStar Health Research Institute, Georgetown University School of Medicine, Washington, DC; Medstar Health, Washington DC, DC; Medstar Health, Washington DC, DC; Uniformed Services University of the Health Sciences, Bethesda, Maryland

## Abstract

**Introduction:**

Burn injury causes a complex pathophysiology resulting in morbidity and mortality often characterized by the development of infectious complications. In health, the microbiota is crucial in host homeostasis and provides numerous benefits. The oral cavity and intestines are important physiologic structures for energy intake where resident microbes participate in metabolic processing. Any potential commonalities in the alterations in oral and gut microbiota after burn have not been previously addressed. Therefore, our study aimed to determine whether severe burns affect the oral and gut microbiota in a consistent pattern during the early stages post-burn.

**Methods:**

Yorkshire swine were placed under anesthesia, and 60% total body surface area burn (n =4) or sham (n =6) injuries were created. Animals were resuscitated with lactated ringer’s based on the Parkland formula. These two cohorts were used to investigate longitudinal microbiome alterations at hours 2, 4, 16, 24, 36, and 48. No antibiotics were given. Shallow shotgun sequencing was performed on DNA isolated from lingual and rectal swabs with downstream α-diversity, β-diversity, and taxonomic analyses performed.

**Results:**

The lingual microbiome had a significantly higher Shannon α-diversity as compared to the gut microbiota in the control (Kruskal-Wallis (KW), *P* = 0.04) and burn (*P* = 0.01) cohorts. There were distinct community differences between the lingual and gut microbiomes based on the Jaccard β-diversity metrics (PERMANOVA, *P* = 0.02). In lingual samples, Shannon diversity significantly increased from baseline by hour 16 in both the control (KW, *P* = 0.009) and burn (*P* = 0.02) groups returning closer to baseline values at hour 48. Jaccard diversity for lingual samples in both groups followed the Shannon diversity trend with significant community shifts observed by hour 16 in controls (PERMANOVA, *P* = 0.011) and burn samples (*P* = 0.032). Bacteroidetes levels increased in lingual samples from baseline to hour 16 in both groups, significantly in the control cohort (Dunnett’s test, *P* = 0.016). In the rectal samples, Shannon diversity decreased from baseline in the control and burn groups to hour 16 although no significant differences were observed. No significant differences in Jaccard β-diversity were seen in rectal samples from the control or burn groups between baseline communities or time points. Similar trends in rectal bacterial composition at the phylum level in both groups were observed with longitudinal non-significant increases in Bacteroidetes and decreases in Firmicutes.

**Conclusions:**

The results suggest that induction and anesthesia cause microbial alterations similar to burn injury during the acute 48-hour time period.

**Applicability of Research to Practice:**

Multi-center whole genome sequencing studies are needed to explore microbial changes post-burn and should include clinical interventions to gain a more thorough understanding of the connection between the oral and gut microbiota.

